# Defining *Brugia malayi* and *Wolbachia* symbiosis by stage-specific dual RNA-seq

**DOI:** 10.1371/journal.pntd.0005357

**Published:** 2017-03-30

**Authors:** Alexandra Grote, Denis Voronin, Tao Ding, Alan Twaddle, Thomas R. Unnasch, Sara Lustigman, Elodie Ghedin

**Affiliations:** 1 Center for Genomics and Systems Biology, Department of Biology, New York University, New York, New York, United States of America; 2 Molecular Parasitology, New York Blood Center, New York, New York, United States of America; 3 Department of Global Health, University of South Florida, Tampa, Florida, United States of America; 4 College of Global Public Health, New York University, New York, New York, United States of America; Case Western Reserve University, UNITED STATES

## Abstract

**Background:**

Filarial nematodes currently infect up to 54 million people worldwide, with millions more at risk for infection, representing the leading cause of disability in the developing world. *Brugia malayi* is one of the causative agents of lymphatic filariasis and remains the only human filarial parasite that can be maintained in small laboratory animals. Many filarial nematode species, including *B*. *malayi*, carry an obligate endosymbiont, the alpha-proteobacteria *Wolbachia*, which can be eliminated through antibiotic treatment. Elimination of the endosymbiont interferes with development, reproduction, and survival of the worms within the mamalian host, a clear indicator that the *Wolbachia* are crucial for survival of the parasite. Little is understood about the mechanism underlying this symbiosis.

**Methodology/ Principle findings:**

To better understand the molecular interplay between these two organisms we profiled the transcriptomes of *B*. *malayi* and *Wolbachia* by dual RNA-seq across the life cycle of the parasite. This helped identify functional pathways involved in this essential symbiotic relationship provided by the co-expression of nematode and bacterial genes. We have identified significant stage-specific and gender-specific differential expression in *Wolbachia* during the nematode’s development. For example, during female worm development we find that *Wolbachia* upregulate genes involved in ATP production and purine biosynthesis, as well as genes involved in the oxidative stress response.

**Conclusions/ Significance:**

This global transcriptional analysis has highlighted specific pathways to which both *Wolbachia* and *B*. *malayi* contribute concurrently over the life cycle of the parasite, paving the way for the development of novel intervention strategies.

## Author summary

Filarial nematodes currently infect millions of people worldwide and represent a leading cause of disability. Currently available medications are insufficient in reaching elimination of these parasites. Many filarial nematodes, including *Brugia malayi*, have an Achilles heel of sorts—that is their obligate symbiotic relationship with the bacteria *Wolbachia*. While it is known that the nematode and the bacteria are co-dependent, the molecular basis of this relationship remains poorly understood. Using deep sequencing, we profiled the transcriptomes of *B*. *malayi* and *Wolbachia* across the life cycle of the parasite to determine the functional pathways necessary for parasite survival provided by the co-expression of nematode and bacterial genes. Defining the mechanisms of endosymbiosis between these two organisms will allow for the exploitation of this relationship for the development of new intervention strategies.

## Introduction

Human filarial infections are currently a leading cause of morbidity in the developing world. Despite the large cost to human health, the chronic and debilitating diseases caused by filarial nematodes remain largely neglected. Two of the most prevalent chronic diseases caused by filaria include lymphatic filariasis, caused by *Wuchereria bancrofti*, *Brugia malayi*, and *Brugia timori*, and onchocerciasis, caused by *Onchocerca volvulus* [1]. Currently 38.5 million people have lymphatic filariasis while 15.5 million people have onchocerciasis, representing in 2015 over 300,000 years lived with disability (YLDs) [2]. While efforts to mitigate the effects of these diseases have been successful in some regions, current medications are insufficient to reach elimination by 2020, particularly in regions of co-endemicity with loasis, caused by the filarial nematode *Loa loa* [3]. Current mass drug administration relies on a small arsenal of drugs, increasing the likelihood of development of resistance, a phenomenon already observed in their veterinary applications [4]. One such drug, Ivermectin, the primary control strategy for onchocerciasis, is unsafe to use in regions where lymphatic filariasis or onchocerciasis are co-endemic with loasis due to the risk of severe adverse effects in individuals heavily infected with *Loa* *loa*.

Most filarial nematodes are hosts for an obligate bacterial endosymbiont, the intracellular bacteria of the genus *Wolbachia*. As the filariae require these bacteria to develop, reproduce and survive in the human host, they represent an attractive target for intervention. The bacteria reside in the lateral cords of the larval and adult nematodes (male and female) as well as in the ovaries and developing embryos of the adult female worms. While the relationship between the nematode and the bacteria is known to be co-dependent, the molecular basis for this relationship remains poorly understood. *Wolbachia* are required for the parasite to reproduce and develop in the mammalian host, while the parasite likely provides amino acids required for bacterial growth [5]. Analyses show significant degradation of the *Wolbachia* genome compared to its free-living relatives, yet it appears to have maintained a number of intact metabolic pathways such as riboflavin, heme, and nucleotide synthesis [5, 6], three pathways that are deficient in the nematode host [7]. As these metabolites are considered essential to all living things, these deficiencies may underlie the symbiotic relationship. Interestingly, in a genome-wide screen for diversifying selection, genes for heme, riboflavin, and nucleotide biosynthesis were found to be under positive selection, again implying they may be integral to the symbiotic relationship [8]. Curiously, however, the recently sequenced *L*. *loa* genome, a *Wolbachia*-free filarial nematode believed to have lost the endosymbiont, also lacks these metabolic pathways and does not appear to have acquired them through horizontal gene transfer [9, 10]. This suggests that filarial worms could also be acquiring these essential metabolites from their mammalian hosts. Thus, the basis of the filaria-*Wolbachia* co-dependency has still not been clarified with the availability of the genomes.

Clearance of *Wolbachia* with the use of antibiotics results in significant apoptosis of filarial germline cells, cells of developing embryos in the female worms, as well as somatic cells of the microfilaria. These effects are non cell-autonomous, meaning cell death is not restricted to cells infected with *Wolbachia* pre-treatment [9]. It is hypothesized that *Wolbachia* are preventing apoptosis by one or both of two possible mechanisms: i) *Wolbachia* are interfering with the host apoptotic program to prevent cell death, and ii) *Wolbachia* secrete some necessary metabolic product(s) that prevent cell death. In this study, we profiled the transcriptomes and inferred co-expression of genes in *Wolbachia* and *B*. *malayi* during the development of male and female worms to identify co-expressed pathways necessary for mediating the endosymbiotic relationship.

## Materials and methods

### Parasites and study design

Parasites were obtained from FR3 where they were isolated and separated by sex from infected gerbils (*Meriones unguiculatus*) at 16 (L4), 30, 42 and 120 days post infection (dpi). Worms were flash frozen and shipped to the New York Blood Center for processing.

### RNA isolation, library preparation and sequencing

*B*. *malayi* worms where homogenized in Trizol (ThermoFisher) using a hand-held pestle in 1.5mL tubes containing the worms. For extraction, 2,000 L4s, 50 male and female juveniles (at 30 dpi and 42 dpi), and 10 male and female adult worms (120 dpi) were used, with two biological replicates for each. Total RNA was extracted by organic extraction using Trizol. A portion of each sample was saved for a DNA extraction while the rest was treated with DNaseI (New England Biolabs). Ribosomal RNA (rRNA) depletion was performed using Terminator (Epicentre), a 5’-phosphate-dependent exonuclease that degrades transcripts with a 5’ monophosphate. Libraries were prepared using the NEBNext Ultra RNA Library Prep Kit for Illumina (New England Biolabs) according to manufacturer instructions. Library quality was assessed using a D1000 ScreenTape Assay (Aligent) prior to sequencing. Library concentrations were assessed using the qPCR library quantification protocol (KAPA biosystems). Libraries were sequenced on the Illumina HiSeq2500 platform with 150bp paired-end reads. To minimize the confounding effects of lane-to-lane variation, libraries were multiplexed and sequenced with technical replicates on multiple lanes. Each developmental stage received an average of 141 million mapped reads.

### Sequencing alignment and differential expression analysis

Read quality was assessed using FastQC (Babraham Bioinformatics). Sequence reads from each sample were demultiplexed and analyzed with the Tuxedo suite of tools [11–13]. Reads were mapped to the annotated *B*. *malayi* (WormBase.org) and *Wolbachia* [6] genome assemblies with Tophat2’s (v2.1.1) Bowtie2-very-sensitive algorithm [11]. The resulting BAM files were then used with Cufflinks (v2.2.1) [11–13] to obtain fragments per kilobase of exon per million fragments mapped (FPKMs) for each of the annotated transcripts and with Cuffnorm [11–13] to obtain normalized FPKMs, normalized for library size. The Tophat2 alignment files were also used to determine differentially expressed genes in both organisms by first using HTSeq (v0.6.1p2) [14] to generate read counts for each gene. Raw read counts were used as input to EdgeR (v3.16.5) [15] to obtain differentially expressed genes between life stage. Genes were determined as significantly differentially expressed using a threshold of *p* <0.05 and a false-discovery rate (FDR) of 5%, standard settings in EdgeR.

### Co-expression analysis

To make the co-expression network and identify the co-expressed gene modules in the symbiosis between *B*. *malayi* and *Wolbachia*, we normalized the gene expression profiles of *B*. *malayi* and *Wolbachia* using Cuffnorm [11–13] and then performed weighted gene correlation network analysis (WGCNA) on the combination of normalized gene expression of *B*. *malayi* and *Wolbachia* using the WGCNA package in R [16]. Hierarchical clustering and dynamic branch cutting were used to identify stable modules of densely interconnected genes. GO term information was downloaded from WormBase.org. Metadata including WSP (*Wolbachia* Surface Protein) and a ratio of *wsp* to *gst* (glutathione-S-transferase) were all integrated into the co-expression network.

### cDNA synthesis and RT-qPCR

To estimate the relative expression of *Wolbachia* genes over different stages of worm development, the DNAse-treated RNA stored in aliquots that were prepared for library preparation and sequencing (see above) was used as a template for cDNA synthesis using the SuperScript III First Strand cDNA Synthesis Kit (Invitrogen). The cDNA was prepared from two biological replicates.

Gene expression was estimated using the standard ‘ΔΔCt’ method. For internal control of *Wolbachia* gene expression, we selected two housekeeping genes (*wBm0291* and *wBm0528*) based on their constitutive expression over the development of the worm according to the RNA-seq data.

### DNA isolation and qPCR

DNA was extracted from *B*. *malayi* worms (the same samples as RNA) by taking the non-organic fraction of trizol/chloroform solutions (see above). DNA was precipitated by ethanol and diluted in water. *Wolbachia* numbers per worm were quantified by qPCR using primers for a *Wolbachia* single-copy gene (*wsp*) as previously described [17].

### Accession numbers

Expression data have been deposited in the Sequence Read Archive (SRA) under Accession number SRP090644.

## Results

### Transcriptome overview

**Fig 1:**
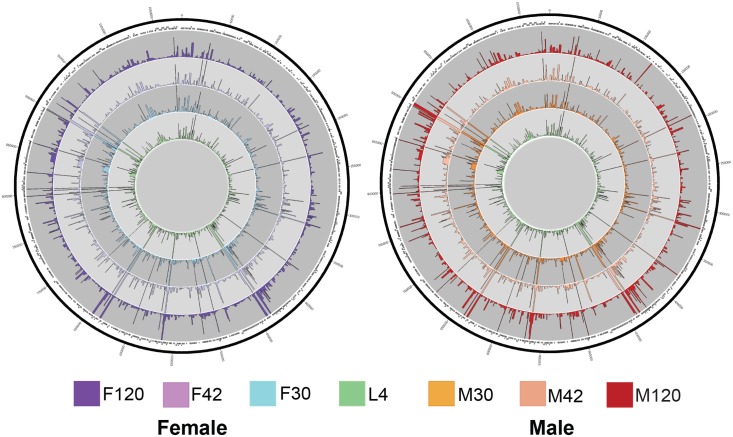
Circos plots of stage-specific *Wolbachia* gene expression. *Wolbachia* expression profiles using normalized FPKMs over development of male and female worms from L4 to 120 days post infection (dpi); genomic location is shown around the perimeter, and the black rectangles represent the CDS.

**Fig 2:**
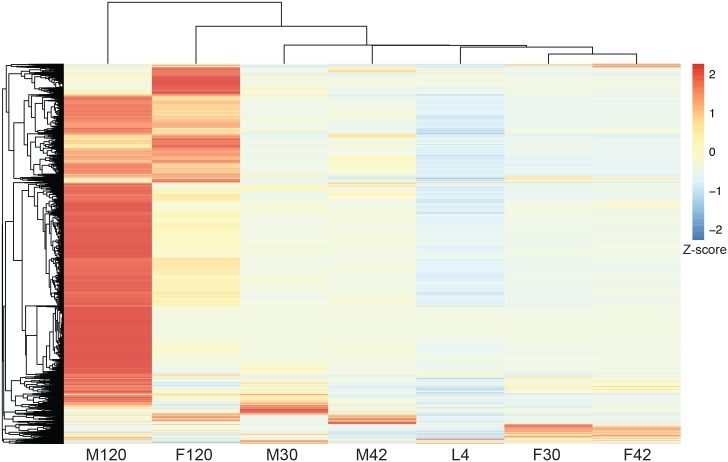
Clustering of stages based on *B*. *malayi* gene expression. Clustering of *B*. *malayi* genes and developmental stages, L4 to 120 days post infection (dpi) Male (M) and Female (F), based on gene expression in normalized FPKMs. Expression was scaled using Z score prior to clustering, with red representing high expression and blue representing low expression. Biological replicates were combined prior to analysis.

To obtain a global view of the transcriptional programs of both *B*. *malayi* and *Wolbachia* concurrently, over the course of worm development from L4 to adulthood, we performed dual RNA-seq. In total over 988 million (or 486 paired-end) RNA-seq reads out of 1.5 billion reads (65.9%) obtained were mapped to the *B*. *malayi* and *Wolbachia* reference genomes (Fig 1: Circos plots for *Wolbachia*). Mapped reads per stage ranged from 77 to 216 million for the *B*. *malayi* genome and 2.1 to 3.7 million for the *Wolbachia* genome (Table 1: Sequencing summary). We found over 96% of *B*. *malayi* gene models to be “expressed” in at least one stage (i.e. a minimum of four cumulative reads across the two independent biological replicates) (Fig 2: Clustering of *Brugia* Expression) (Table 1: Sequencing Summary). Expression in *B*. *malayi* was dominated by sex-biased gene expression, with the 120 dpi adult male and females expressing the most genes at the highest expression levels (Fig 2). In *Wolbachia*, 85% of gene models were classified as expressed (Table 1: Sequencing Summary). Sequence reads from technical replicates—i.e. the same library sequenced on different lanes of the HiSeq—were combined per biological replicate as they contained the same insert size distribution. Using multidimensional scaling analysis, we clustered biological replicates for each *B*. *malayi* developmental stage (S1a Fig: *B*. *malayi* MDS). All biological replicates clustered closely to each other with the exception of the F42 replicates, where F42b clustered more closely with the F30 replicates than with F42a. However, when we clustered biological replicates for *Wolbachia* reads, F42a and F42b clustered closely together (S1b Fig: *Wolbachia* MDS). It is thus unlikely that the observed *B*. *malayi* disparate clustering for that stage is due to the mislabeling or contamination of the sample and more an effect of natural population variation. Generally, clustering of the stages indicates good reproducibility of the biological replicates, with the 30 dpi samples of both males and females clustering more closely with the mixed-gender L4s than to each other, followed by the 42 dpi samples. As expected, the 120 dpi male and female samples are found to be the most different from each other than the other samples from earlier life stages.

**Table 1:** Dual RNA-seq sequencing summary. The table shows the total reads sequenced and mapped in each biological replicate at each developmental stage, L4 to 120 days post infection (dpi) males (M) and females (F), lower case a and b refer to separate biological replicates.

Sample	Total reads (million)	Left mapped reads (million)	Right mapped reads (million)	Total mapped reads (million)	% Mapped reads	Stage total reads mapped to Brugia (million)	% Brugia Genes Expressed	Stage total reads mapped to Wolbachia (million)	% Wolbachia genes expressed
**L4a**	104	24	23	47	45	94	69	2	66
**L4b**	91	25	24	49	54				
**F30a**	82	20	19	39	48	87	75	3	61
**F30b**	101	25	25	50	50				
**F42a**	172	75	73	149	87	185	78	3	60
**F42b**	74	20	19	39	53				
**F120a**	195	85	81	166	85	212	94	4	84
**F120b**	105	25	25	50	48				
**M30a**	57	16	16	32	56	75	78	2	62
**M30b**	83	23	23	46	55				
**M42a**	158	71	68	139	88	206	82	3	62
**M42b**	119	35	35	70	59				
**M120a**	90	40	39	78	87	110	91	3	65
**M120b**	56	17	17	34	61				
**Total**	1486	502	487	989		969	97	19	85

To validate the use of RNA-seq for the purpose of transcriptional analysis, seven *Wolbachia* genes, with ten pair-wise comparisons, were selected for qRT-PCR analysis of their relative expression. Four of the genes (*wsp*, *Hsp90*, *DnaK*, and *GroEL*), with seven significant pair-wise comparisons, were chosen based on the criteria that they were found to be significantly differentially expressed and had over 50 read counts per stage. We also included three genes (*RibA*, *HemA*, and *AfuA*) that were constitutively expressed, based on an FDR of 1 in EdgeR, which indicates that they were the least likely to be differentially expressed. We observed a spearman correlation between the qRT-PCR and RNA-seq results of 0.987 and a *p*-value < 2.2e-16 (S2 Fig: qPCR validation graph).

### Differential expression is dominated by female stages

Differential expression in *B*. *malayi* was dominated by sex-biased gene expression, as previously observed [18], with the largest number of sex-biased genes at 120 dpi, with 2,753 genes showing male bias, and 3,109 showing female biased expression (S1 Table: *Brugia* DE Female, S2 Table: *Brugia* DE Male, and S3 Table: *Brugia* DE Male to Female). We find that 82% of the genes previously determined to be significantly up-regulated in adult male worms and 79% of the genes significantly up-regulated in females worms [18], were, in our new data set, also up-regulated in male worms (M120) or female worms (F120), respectively, as compared to worms of the opposite sex. This shows good reproducibility between the two studies, although it should be noted that many additional genes were found to be differentially expressed between F120 and M120 worms in the new data set. This is likely due to the use of biological and technical replicates, as well as a higher depth of coverage.

To uncover the role *Wolbachia* may play in worm development, we analyzed differentially expressed *Wolbachia* genes in male and female worms at each developmental stage. Pair-wise differential expression analysis was performed using EdgeR, after removing all genes with zero expression in two or more samples per comparison. The percentage of differentially expressed genes in any pair-wise comparison ranged from 0–4.8% of *Wolbachia* genes expressed (Table 1).

**Fig 3:**
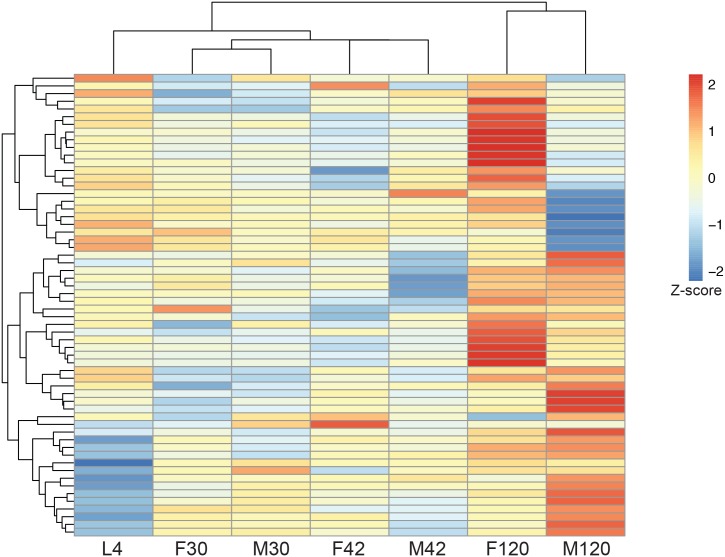
Clustering of stages and *Wolbachia* DE genes. Hierarchical clustering of *Wolbachia* DE genes and developmental stages, L4 through 120 days post infection (dpi) male (M) and female (F), based on gene expression in normalized FPKMs. Expression values were scaled prior to clustering using a Z score calculation, with red representing high expression and blue representing low expression. Biological replicates were combined prior to clustering.

We identified a total of 62 differentially expressed (DE) *Wolbachia* genes across a single or multiple pair-wise comparisons (Fig 3: Clustering of *Wolbachia* DE genes). The largest number of *Wolbachia* DE genes (34 genes) is in the females from 42 dpi (F42) to 120 dpi (F120), while there are no DE genes in the 30 to 42dpi comparisons in both males and females (Table 2: DE summary). In comparing stages between genders, there were 17 DE genes between females and males at 120 dpi (F120 and M120), and no genes differentially expressed between both sexes at 30 or 42 dpi. Comparisons between female stages consistently resulted in more DE genes than did comparisons between male stages (Table 2: DE summary): 40 *Wolbachia* genes were determined as DE over the course of female growth, but were absent in any male comparisons. Because these genes appear to be differentially regulated during female worm development only, they are potentially required for female-specific processes known to be dependent on *Wolbachia* infection, such as maturity of female gonads and germline development, as well as embryogenesis. The ten *Wolbachia* genes determined as DE in both males and females represent potential expression in the lateral cords, required for the development of both the male and female germlines. Twelve *Wolbachia* genes were determined as DE in males only (S4 Table: *Wolbachia* DE genes).

**Table 2:** Summary of differential expression. The table shows the results of the EdgeR pair-wise comparisons between developmental stages, L4 to 120 days post infection (dpi) in males (M) and females (F). Genes are said to be up-regulated if they are higher in the second developmental stage in the comparison, and down-regulated if they are lower in the second developmental stage.

Stage	L4-F30	F30-F42	F42-F120	F30-F120	L4-M30	M30-M42	M42-M120	M30-M120	F30-M30	F42-M42	F120-M120
**Wolbachia Genes**	682	673	728	728	682	681	689	691	673	678	734
**Total DE Wolbachia**	7 (1.0%)	0	34 (4.7%)	27 (3.7%)	3 (0.4%)	0	7 (1.0%)	4 (0.6%)	0	0	17 (2.3%)
**Wolbachia Up**	1	0	17	15	0	0	3	3	0	0	12
**Wolbachia Down**	6	0	17	12	3	0	4	1	0	0	5
											
**Brugia Genes**	9,269	9,431	10,424	10,295	9,384	9,929	10,386	10,356	9,517	9,892	10,643
**Total DE Brugia**	1,129 (12.2%)	13 (0.1%)	1,350 (13.0%)	4,113 (40.0%)	930 (9.9%)	2,036 (20.5%)	3,101 (29.9%)	3,580 (34.6%)	1,243 (13.1%)	456 (4.6%)	5,281 (49.6%)
**Brugia Up**	474	12	805	1,995	538	1,118	1,398	1,535	697	265	2,817
**Brugia Down**	655	1	545	2,118	392	918	1,703	2,045	546	191	2,464

### Up-regulated *Wolbachia* genes during female development are enriched for chaperone function, energy production and translation

The pattern of *Wolbachia* differential expression in the female stages was dominated by chaperone protein expression (Table 3: DE expression of Chaperones). Integral membrane proteins, translation, antioxidants, oxidative phosphorylation, DNA replication, and peptidase function were also highly represented (S4 Table). GO-term enrichment analysis of *Wolbachia* DE genes during female development using Fisher’s exact test revealed an enrichment of GO terms associated with chaperone function including protein-folding and unfolded protein-binding (Table 3). We detected seven genes with chaperone function that were significantly up-regulated in F30 as well as in F42, four of which are also significantly up-regulated in F120 as compared to M120. The four genes found to be significantly up-regulated in all female stages include *wBm0350 groEL* and co-chaperonin *wBm0349 groES*, which work in complex as an integral part of several stress responses in bacteria, including the oxidative stress response, where they recover oxidized proteins [19–23]. Also highly up-regulated in all female stages is the molecular chaperone *wBm0533 grpE*, shown to assist in protein refolding during oxidative stress, as well as *HslU*, a subunit of an ATP-dependent protease with chaperone function [24, 25]. Among the chaperones significantly up-regulated in the F30 and F42 stages, but not F120, are *DnaK* and *DnaJ*, another molecular chaperone system shown to be required for cell division in bacteria as well as for resistance to heat shock [23].

**Table 3:** DE expression of chaperones. The table shows the differential expression of genes encoding chaperone proteins over the development of the parasite, L4 to 120 days post infection (dpi) males (M) and females (F). The stage at which the gene is up-regulated is listed, and the stage at which it was compared to is in parentheses.

Gene	Differential Expression	Description	logFC	logCPM	PValue	FDR
Wbm0138	Upregulated in F30 (F120)	Heat shock protein 90	-1.30	6.72	8.25E-06	6.01E-04
Wbm0350	Upregulated in F30 (F120)	Molecular chaperone GroEL	-1.48	9.79	4.90E-16	1.78E-13
Wbm0785	Upregulated in F30 (F120)	Molecular chaperone DnaJ	-1.51	6.38	3.13E-06	3.20E-04
Wbm0533	Upregulated in F30 (F120)	Molecular chaperone GrpE (heat shock protein)	-1.85	4.85	4.06E-04	1.65E-02
Wbm0495	Upregulated in F30 (F120)	Molecular chaperone DnaK	-1.87	9.10	1.09E-13	2.65E-11
Wbm0349	Upregulated in F30 (F120)	Co-chaperonin GroES	-1.95	7.55	2.83E-11	5.14E-09
Wbm0723	Upregulated in F30 (F120)	ATP-dependent protease ATP-binding subunit HslU	-1.99	6.41	8.21E-08	1.19E-05
Wbm0349	Upregulated in F42 (F120)	Co-chaperonin GroES	-1.72	7.60	2.07E-05	1.51E-03
Wbm0785	Upregulated in F42 (F120)	Molecular chaperone DnaJ	-1.94	6.32	1.60E-07	1.66E-05
Wbm0495	Upregulated in F42 (F120)	Molecular chaperone DnaK	-2.13	9.06	2.42E-13	8.81E-11
Wbm0138	Upregulated in F42 (F120)	Heat shock protein 90	-2.14	6.57	1.42E-10	3.46E-08
Wbm0533	Upregulated in F42 (F120)	Molecular chaperone GrpE (heat shock protein)	-2.20	4.82	7.75E-05	4.34E-03
Wbm0350	Upregulated in F42 (F120)	Molecular chaperone GroEL	-2.34	9.63	1.74E-19	1.27E-16
Wbm0723	Upregulated in F42 (F120)	ATP-dependent protease ATP-binding subunit HslU	-2.39	6.37	2.35E-09	3.43E-07
Wbm0350	Upregulated in F120 (M120)	Molecular chaperone GroEL	-1.75	9.92	1.80E-04	1.62E-02
Wbm0723	Upregulated in F120 (M120)	ATP-dependent protease ATP-binding subunit HslU	-1.88	6.61	9.17E-04	3.99E-02
Wbm0533	Upregulated in F120 (M120)	Molecular chaperone GrpE (heat shock protein)	-2.09	4.97	7.59E-04	3.98E-02
Wbm0349	Upregulated in F120 (M120)	Co-chaperonin GroES	-2.50	7.64	1.63E-07	1.19E-04

Among the *Wolbachia* DE genes in adult female worms (F120) are a number of genes involved in combating oxidative stress. *wBm0439* coenzyme Q-binding protein, an antioxidant, is significantly up-regulated from F30 to F120 as well as from F42 to F120. We also find *wBm0220 SodA*, a superoxide dismutase, to be significantly up-regulated from F42 to F120. *SodA* catalyzes the conversion of superoxide radicals to hydrogen peroxide and oxygen and is known to be essential in combating oxidative stress [19]. Additionally, we detect significant upregulation from F42 to F120 of *wBm0674*, a malic enzyme responsible for the interconversion of L-malate and pyruvate. This reaction is essential for maintaining cellular pools of NADPH, required for a number of downstream processes including reducing oxidative stress [26, 27].

A number of genes determined as DE during female development are involved in energy production. It is hypothesized that a key mechanism of the *Wolbachia*-host symbiosis is aerobic energy production by the bacteria for the worm [28–30]. NADH dehydrogenase subunit B (*wBm0242*), which is involved in oxidative phosphorylation, is significantly up-regulated in F30 as compared to L4s. ATP synthase subunit C, which creates ATP using a proton gradient, is also up-regulated in F120 as compared to F30, and in M120 as compared to F120. Two proteins involved in iron-sulfur cluster formation are also up-regulated in F120 as compared to earlier female stages: *wBm0756*, an iron-sulfur cluster assembly scaffold protein, and *wBm0448*, a succinate dehydrogenase flavoprotein. Iron-sulfur clusters are essential co-factors for respiratory chain proteins involved in ATP production [28].

Several glycolytic enzymes were significantly up-regulated in F120 and M120 as compared to earlier stages, including transaldolase *(wBm0686)*, an enzyme linking the pentose phosphate pathway to glycolysis, and *wBm0097*, a fructose-bisphophase aldolase. Notably, the *Wolbachia* genome lacks two glycolytic enzymes (6-phosphofructokinase and pyruvate kinase) likely rendering the glycolytic pathway defective. *Wolbachia* may therefore depend on products from the *B*. *malayi* glycolytic cycle such as pyruvate, as well as TCA cycle intermediates derived from amino acids. Accordingly, *wBm0207* pyruvate dehydrogenase, which transforms pyruvate into acetyl-CoA that can then be used in the citric acid cycle, was differentially expressed during female development. Correspondingly, in *B*. *malayi* we see an upregulation of *Bm5241* in F120, involved in the glycogen catabolic process. We also see differential expression of *wBm0384*, an extracellular metalloprotease potentially involved in the breakdown of filarial peptides for amino acids during female development [6]. Additionally we see the two Zn-dependent peptidases cluster with most of the TCA cycle enzymes based on expression. Together with the up-regulation of pyruvate dehydrogenase, this co-expression suggests an increased dependence on *B*. *malayi* products for energy production.

Another functional category represented in the up-regulated genes in F120 as compared to younger female stages is that of DNA replication. DNA polymerase III gamma/tau subunit (*wBm0434*) and *recJ* (*wBm0124*), a single-stranded DNA-specific exonuclease involved in single-strand break repair, and DNA/RNA helicase (*wBm0708*), required for both DNA replication and transcription, are significantly up-regulated at this stage, as is the RNA polymerase omega subunit (*wBm0387*), indicating an increase in transcription during this developmental stage. We determined two ribosomal proteins, S4 and S15, to also be significantly up-regulated at F120. The ribosomal protein S4 is essential for protein synthesis through its function in RNA binding, leading to fewer errors, while S15 plays an essential role in the assembly of the central domain of the small ribosomal subunit [31]. Our observations are consistent with findings in the *Wolbachia* populations in the gonads of *O*. *ochengi* showing differential regulation of *Wolbachia* genes required for DNA replication and translation, including ribosomal proteins in the germline [29]. Genes involved in lipid II/ peptidoglycan biosynthesis (*wBm0493*, *metC*, and *wBm0492*, *murE*) and *wBm0490*, a protein shown to interact with *ftsH*, a gene required for cell division, were also found to be significantly up-regulated in F120.

Among the genes differentially expressed over the course of female development are three peptidases including *wBm0384*, an extracellular metallopeptidase unique to the *Wolbachia* of *B*. *malayi*, that are potentially involved in the breakdown of filarial peptides for amino acids [6]. The other two genes, *wBm0772* and *wBm0552*, encode ATP-dependent protease subunits.

Genomic analysis revealed that *Wolbachia* has maintained the biosynthetic pathways for purines and pyrimidines while *B*. *malayi* has not, suggesting that *Wolbachia* are potentially provisioning nucleotides to their filarial hosts, especially during times of increased need. In accordance with this hypothesis, *wBm0443* guanosine monophosphate synthase, an essential enzyme in *de novo* purine biosynthesis, is differentially expressed during female worm development. Additionally, we find *wBm0255* amidophosphoribosyltransferase, also involved in *de novo* purine biosynthesis, to be differentially expressed in both males and females. This supports a potential role of nucleotide production by *Wolbachia* in the lateral cords in male and female worm development.

It was proposed that *Wolbachia* might be inhibiting apoptosis in the worm host through the manipulation of the host apoptotic pathway [9, 29]. While very little is understood about how this may be occurring, we find significant up-regulation of three genes putatively involved in the manipulation of the apoptotic pathways in F120. One such gene is *wBm0152*, a *Wolbachia* surface protein, shown to inhibit apoptosis of purified human polymorphonuclear cells *in vitro* [32]. We also find significant up-regulation of *wBm0296*, an ankyrin repeat-containing protein, hypothesized to be an effector protein of the Type-IV secretion system (T4SS) able to mediate interactions with the host cells as they are for other intracellular bacteria [33–35]. Lastly, *wBm0490*, a gene with high homology to a bax-inhibitor in the *Wolbachia* of *Drosophila*, is significantly up-regulated in F120. Manipulation of the host apoptotic pathway through the expression of bax-inhibitors is believed to be responsible for the suppression of apoptosis of host cells by the obligate intracellular bacteria, *Chlamydia trachomatis* [36].

### Constitutive expression of biosynthetic pathways in *Wolbachia* that are missing in *B*. *malayi*

**Fig 4:**
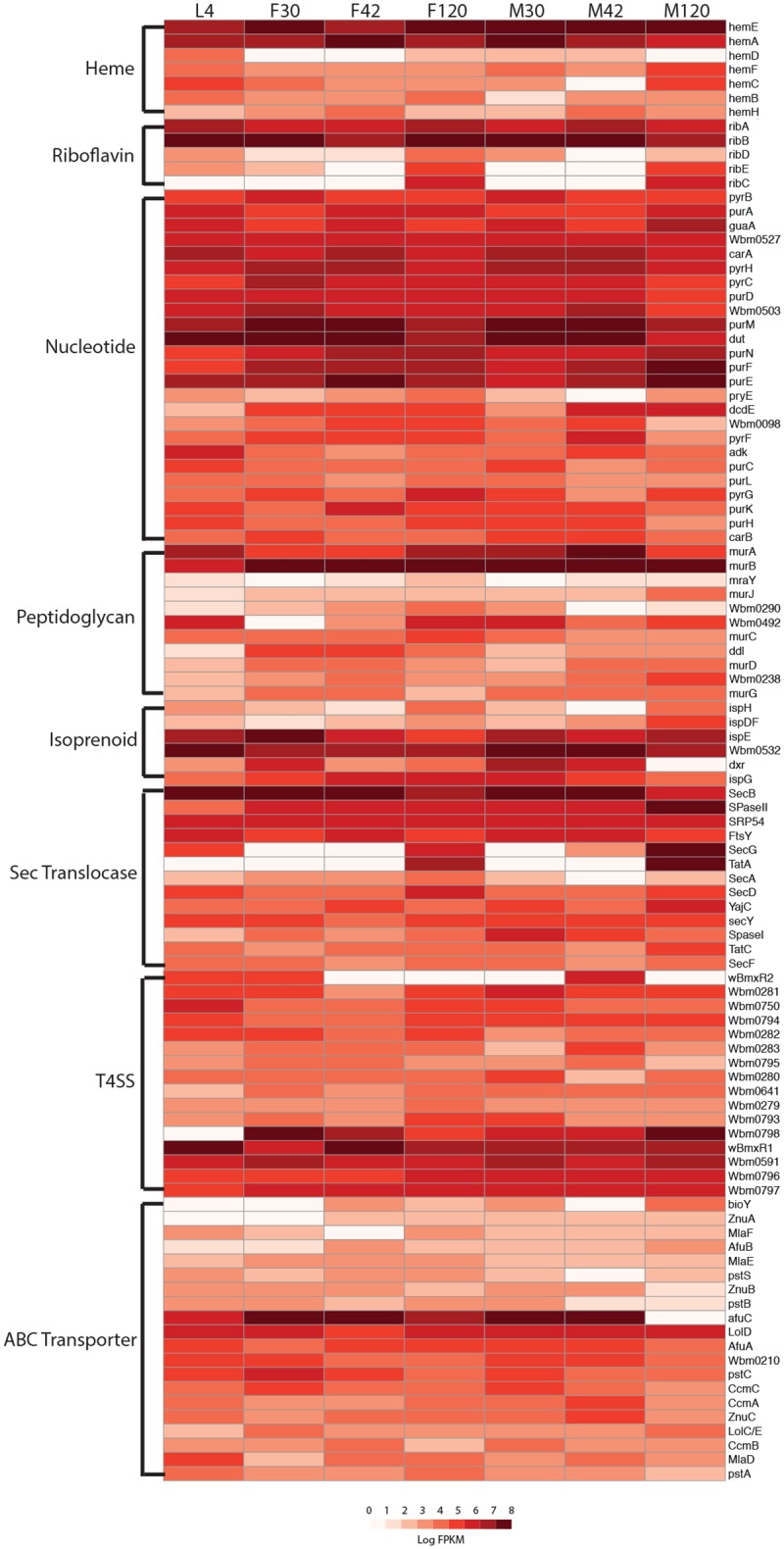
Heatmap of gene expression of pathways of interest. Expression, in log FPKMs, is plotted over the development of the nematode, L4 to 120 days post infection (dpi) male (M) and female (F), by pathway for heme, riboflavin, FAD, peptidoglycan, and isoprenoid biosynthesis as well as expression of the main secretion systems in *Wolbachia* including Sec translocase, Type IV secretion system, and ABC transporters.

The expression in *Wolbachia* of three biosynthetic pathways (heme, riboflavin, and FAD) potentially important for symbiosis as well as *Wolbachia* transporters, were examined in each developmental stage (Fig 4: Heatmap of gene expression of pathways of interest). Common to all *Wolbachia* genomes sequenced thus far is the presence of nearly all genes necessary for the synthesis of the iron-containing cofactor, heme, except for *hemG*, which is missing in many heme-producing bacteria [6, 28, 37]. Heme is an essential cofactor for cytochromes, peroxidases, and catalases, which are involved in a number of critical cellular processes including oxidative phosphorylation and electron transport. Heme is a co-factor for peroxidases essential for molting and might possibly also be a co-factor for steroids involved in molting of filarial parasites [28, 38]. Unlike what was shown for *O*. *ochengi* where very low expression of the heme biosynthetic pathway in adult tissues was detected [29], we found all *Wolbachia* genes involved in heme biosynthesis to be expressed in all sampled stages, with the highest expression at the L4 and F120 stages. Additionally, we find constitutively high expression of iron ABC transporters responsible for importing iron into the bacterial cell as well as heme ABC-transporters responsible for transporting heme from the bacterial cystoplasm into the periplasmic space and potentially involved in the transport of heme into the cytoplasm of the filarial host cell.

Unlike *Rickettsia*, *Wolbachia* has maintained the ability to synthesize both riboflavin and FAD [6]. As riboflavin biosynthesis has been lost in *B*. *malayi*, it was hypothesized that *Wolbachia* were provisioning this cofactor to their filarial hosts. In support of this hypothesis we found that *wBm0416*, involved in FAD biosynthesis, as well as *RibA* and *RibB* are constitutively highly expressed across all stages of worm development. *RibA* is a bifunctional enzyme that catalyzes the first two essential steps in riboflavin biosynthesis, and is co-regulated with the T4SS [39]. F120 is the only stage in which we find all genes in the FAD/ riboflavin biosynthetic pathways to be classified as expressed, and at particularly high levels.

The Sec translocase system is responsible for the majority of protein trafficking across the bacterial cytoplasmic membrane into the periplasm with the use of ATP [40]. *SecY* is a transmembrane protein constituting the core of the protein-translocating complex. *SecY* was constitutively highly expressed across all stages of the life cycle. *SecG* associates with *SecY* to form a heterotrimeric complex. While not necessary for general function of the system, *SecG* has been shown to facilitate transport at low temperatures (20°C), or when the proton-motive-force is reduced [41, 42]. We find expression of secG only in L4, M42, F120, and M120 where we see particularly high expression. While *Wolbachia* lack the tatB gene, part of the Sec-independent twin arginine translocation (Tat) protein system present in most bacteria, they do maintain *TatA* and *TatC* genes. Thus, as in other alpha-proteobacteria, it is likely that this system is still functional [43]. *TatA* was highly expressed in F120 and M120 exclusively, while *TatC* was expressed constitutively across all stages. Secretion in *Wolbachia* requires not only translocation into the periplasmic space by either the Sec or Tat systems, but transport across the outer membrane as well. This is accomplished by the T4SS, a leader-peptide independent mechanism for transporting effector proteins and virulence factors found in many pathogenic and endosymbiotic bacteria [35, 44–46]. We find constitutive expression of nearly all genes in this pathway at most stages except for *wBm0798* in L4.

ATP-binding cassette transporters (ABC transporters) are composed of two transmembrane domains and two cytoplasmic ATP-binding domains. They are involved in the uptake of a variety of nutrients and the extrusion of drugs and metabolites [47]. As previously mentioned, we saw constitutive expression of all four heme ABC transporters encoded in the *Wolbachia* genome, as well as two lipoprotein transporters. Constitutive expression of the lipoprotein transport system *LolCDE* is required to export lipoproteins to the outer membrane [48]. Lipoproteins have been shown to be agonists of inflammatory pathogenesis in lymphatic filariasis, recognized by the *TLR-2* and *TLR-6* in the human host [49]. Correspondingly, we see significant up-regulation in adult female worms of the *wBm0152* peptidoglycan-associated lipoprotein-like outer membrane protein shown to be localized to numerous sites on the bacterial membrane [50]. We also find constitutively high expression of two phosphate transporters, potentially required for importing phosphate for nucleotide production. Experiments in *L*. *sigmodontis* show that when *Wolbachia* is depleted with tetracycline, expression of a filarial phosphate transporter is significantly increased to compensate for the decrease in *Wolbachia*-produced nucleotides that are essential for worm embryogenesis and survival [51].

### Co-expressed nematode and bacterial genes

**Fig 5:**
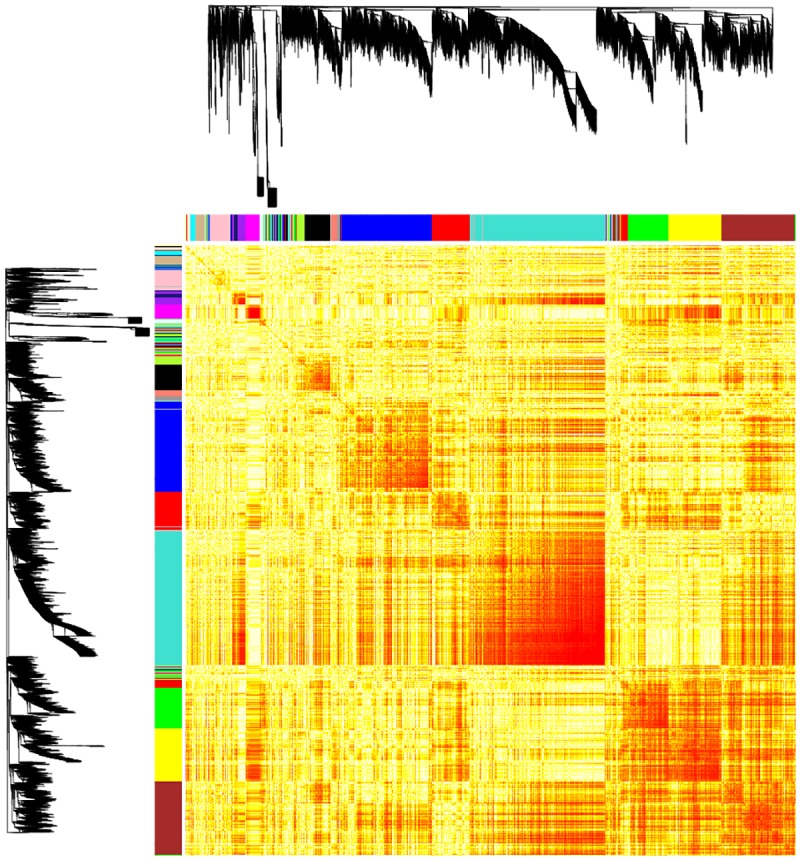
The co-expression network for *B*. *malayi* and *Wolbachia*. The network heatmap plot of network connectivity for *B*. *malayi* and *Wolbachia* calculated using weighted gene correlation network analysis (WGCNA). The black branches show the hierarchical clustering dendrograms, which were assigned to clusters using dynamic tree cutting to identify modules of co-expressed genes, shown as colored bars. High co-expression interconnectedness is indicated by increasingly saturated orange and red coloring. Modules correspond to groups of highly interconnected genes.

To determine which genes were being co-expressed between *B*. *malayi* and *Wolbachia*, we built a co-expression network for the two organisms using WGCNA (Fig 5: The co-expression network for *B*. *malayi* and *Wolbachia*). WGCNA is a well-established method by which expression data and trait data are integrated to identify co-expressed pathways. We used hierarchical clustering and dynamic cutting to determine modules of co-expression. A module is a cluster of interconnected genes with high correlation based on their expression profiles (S5 Table: Summary of module membership). GO term enrichment of each resulting module was performed to determine which modules were biologically significant (S6 Table: GO term Enrichment). To identify modules of gene co-expression with the most interest based on the symbiotic interaction of the two organisms, we evaluated the correlation of each module to a measure of *Wolbachia* population, *wsp/gst*. We found three modules that had the highest negative correlations with the *wsp/gst* ratio (brown, -0.57 *p*-value = 0.03; yellow and green, -0.65 *p*-value = 0.01). A fourth module had the highest positive correlation with the *wsp/gst* ratio (salmon, 0.63 *p*-value = 0.02). Because the F120 samples have the lowest *wsp/gst* ratio ratio due to the large size of the female worms, we determined that the three modules with the highest negative correlation were indicative of adult female gene expression.

The green module contains 2,312 *B*. *malayi* genes and 28 *Wolbachia* genes (S5 Table); it shows an enrichment of DE genes that are up-regulated in the adult females, with a *p*-value < 2.2e-16. The most significantly enriched GO terms in the green module include intracellular signal transduction, proteolysis, transmembrane transport, cyclic nucleotide biosynthetic process, cysteine-type peptidase activity, and mitochondrion. Interestingly, this module also contains the cathepsin-like cysteine protease *Bma-cpl-6* found to be involved in development and embryogenesis in the worm as well as in *Wolbachia* expansion (S6 Table) [52]. The yellow module contains 2,782 *B*. *malayi* genes and 39 *Wolbachia* genes and shows an enrichment of GO terms that include the regulation of transcription, oxidoreductase activity, sequence-specific DNA binding, and cell redox homeostasis. Interestingly, the co-expressed *Wolbachia* genes in this module include six of the seven chaperones found to be differentially expressed during female development as well as a gene involved in redox sensing and two genes involved in cytochrome c biogenesis. Cytochrome c is known to play a role in the electron transport chain and cell apoptosis, as well as being an antioxidative enzyme by removing superoxide and hydrogen peroxide. The brown module contains 4,148 *B*. *malayi* genes and 64 *Wolbachia* genes and shows a significant under-representation of DE genes (*p*-values < 2.2e-16) in F120 as compared to F42. As one of the modules that are the most highly negatively correlated to the *Wolbachia* population, the brown module contains a number of genes implicated in the host/endosymbiont relationship including genes involved in riboflavin and nucleotide biosynthesis (S5 Table). Among the most enriched GO terms in the brown module are metabolism and transport. Co-expressed *Wolbachia* genes include a number of genes also involved in transport, such as three ABC transporters, including an iron importer, a general permease exporter, and a polyamine transporter. Among the many *B*. *malayi* genes involved in transport co-expressed in this module is *Bm4941*, one of three genes in the genome predicted to have nucleoside transmembrane transporter activity based on protein domain information. In the brown module, we find the GO term glycogen catabolic process (GO:0005980) to be significantly enriched, as well as the genes *Bm5241* in *B*. *malayi* and *wBm0207* in *Wolbachia*, previously mentioned in relation to pyruvate metabolism, to be co-expressed in this module. Among the *Wolbachia* genes co-expressed in this module are four genes involved in *de novo* purine and pyrimidine biosynthesis. Also in the brown module are two *Wolbachia* genes in the riboflavin biosynthesis pathway, including *RibA*, which are co-expressed with a number of *B*. *malayi* riboflavin or flavin-requiring proteins. Another set of enriched GO terms in the brown module includes DNA repair and replication, a function also represented with the co-expressed *Wolbachia* genes that include five genes involved in DNA replication and repair, including ribonucleotide reductase, an enzyme integral in controlling the rate of DNA synthesis [53]. Correspondingly, two genes involved in cell division in *Wolbachia* are co-expressed in this module. Interestingly, the salmon module, the module with the highest positive correlation to *Wolbachia* population, is enriched for GO terms related to regulation of apoptosis, response to oxidative stress, oxidase activity, and heme binding.

## Discussion

The dual transcriptional profiling that we performed revealed potential stage-specific requirements from *Wolbachia* during filarial development and embryogenesis. Differentially expressed *Wolbachia* genes during the course of female development generally fell into the functional categories of chaperone function, energy production, nucleotide biosynthesis, DNA replication, and anti-oxidative defense. These categories include genes that are likely to be required for specific developmental processes, including germline and embryonic development in the nematode and *Wolbachia* invasion of the gonad. Similar studies have been performed on the filarial nematode *Dirofilaria immitis*, or dog heartworm, and its *Wolbachia* endosymbiont, *w*Di [30, 54]. While the first study found no *w*Di genes differentially expressed between adult male and female worms, a second tissue-specific study reported differentially expressed *w*Di genes in the uterus and female body wall. Our findings including *w*Bm genes differentially expressed over the course of female development correspond to those *w*Di genes found to be up-regulated in the uterus, including multiple genes encoding ribosomal proteins, DNA replication and repair machinery, a tRNA synthetase, and a component of the purine biosynthetic pathway. This indicates that the role *Wolbachia* play in certain life stages of the filaria may be well conserved.

It has been proposed that the ability of *Wolbachia* to perform aerobic respiration and metabolize iron whilst responding to oxidative stress may be an essential mechanism of the endosymbiotic relationship with filarial worms [28, 29]. Studies in *Litomosoides sigmodontis*, for example, have found that targeting *Wolbachia* with antibiotics resulted in the up-regulation of components of the mitochondrial respiratory chain [55]. Experiments in *O*. *ochengi* showed that worms treated with antibiotics lose motility and that *Wolbachia* density in infected cells greatly exceeded that of mitochondria [56]. These results point to the potential ATP provisioning by *Wolbachia* to the filarial host [56]. The up-regulation of genes involved in the ATP transport chain and in iron-sulfur cluster formation—which are essential co-factors for respiratory chain proteins in ATP production—provide support for this hypothesis but it is difficult to prove. Alternatively, the up-regulation of ATP production could be required for the increased propagation of *Wolbachia* at this stage.

The potential production of ATP by *Wolbachia* for its filarial host likely contributes to oxidative stress of their cellular environment by generating ROS as by-products of aerobic metabolism. Consistent with this hypothesis, many genes that encode proteins known to be involved in combating oxidative stress were highly differentially expressed during female development, including chaperone proteins and a gene involved in single-strand break repair, a potential consequence of an increasingly oxidative environment. A number of these chaperone proteins have been shown to be part of the oxidative stress response in bacteria and to maintain their stability under oxidative conditions [57, 58]. It was proposed that overexpression of *groEL* is an important adaptation allowing for the obligate intracellular lifestyle of *Wolbachia* within a cytoplasmic vesicle [59, 60]. We find that these requirements appear to be of special importance during female development, potentially as a consequence of the up-regulation of oxidative phosphorylation. Chaperones *GroEL*, *HSP60*, and *DnaK* were found to be among the proteins with the most abundant peptide counts in proteomic analysis of *B*. *malayi* in the adult stages of the worm [61]. This study did not however look at abundance over the course of worm development, L4 through 30–42 dpi. The chaperone *HslU* forms a complex with the peptidase *HslV*, which was also found to be significantly up-regulated in the F30 and F42 stages. *HslV*, or *HSP20*, has additionally been shown to be involved in bacteria-host interactions in *Helicobacter pylori* [62]. Interestingly, the *DnaJ/K* chaperones are among the *Wolbachia* genes found to be inserted in the nuclear genomes as well as expressed by the *Wolbachia*-free filariae *A*. *viteae* and *O*. *flexuosa* [63].

While many endosymbionts and parasites, including *B*. *malayi*, as well as members of the *Rickettsia* genus have lost the pathways for *de novo* purine and pyrimidine synthesis, *Wolbachia* has maintained these biosynthetic pathways. Additionally, *Wolbachia* was shown to lack the ADP/ATP translocases used by other endosymbionts, including the parasitic *Rickettsia* and *Chlamydia*, and the mutualist *Buchnera*, to scavenge for nucleotides from the host [8]. These observations, combined with the evidence for positive selection on genes in this pathway suggest that *Wolbachia* produce nucleotides not only for internal consumption but also for the host at times when the requirement for DNA synthesis is particularly high, such as during oogenesis and embryogenesis [6, 8]. During the mitotic proliferation of the *B*. *malayi* oogonia, *Wolbachia* divides rapidly, requiring increased expression of the replication machinery [64]. Correspondingly, we found in F120 significant up-regulation of the *Wolbachia* DNA replication machinery and genes involved in transcription and translation.

In our quest to capture the basis of the endosymbiotic relationship between *B*. *malayi* and *Wolbachia*, we looked at the expression of genes that are part of biosynthetic pathways in *Wolbachia* that are missing in *B*. *malayi*. The absence of the heme, riboflavin and FAD biosynthetic pathways in filaria led to the hypothesis that *Wolbachia* could be providing these to the filarial host. Evidence for this, however, remains elusive. Several studies have shown that inhibitors of the heme biosynthetic pathway such as 5-aminolevulinate (ALAD) and N-methyl mesoporphyrin (NMMP) have adverse effects on *B*. *malayi*, causing a marked reduction in motility [38]. While this suggests a role for *Wolbachia* in provisioning heme, adverse effects were also observed on *C*. *elegans*, which also lack the heme biosynthetic pathways and are *Wolbachia*-free, suggesting non-specific effects. We found constitutive expression of all genes in the heme biosynthetic pathway of *Wolbachia* as well as of heme ABC transporters at almost all sampled worm stages, with the highest expression at the L4 and F120 stages. This suggests the importance of heme synthesis and transport in the symbiotic relationship at these stages [29]. However, it remains unknown if or how *B*. *malayi* might receive heme from its bacterial endosymbiont. Examination of the riboflavin and FAD biosynthetic pathways in *Wolbachia* revealed F120 as the only stage in which all genes are highly expressed, suggesting an increased need in adult females for riboflavin and FAD for embryogenesis. This finding is consistent with the observation that when adult worms are grown in the presence of doxycycline, causing severe adverse effects in embryogenesis, supplementation with riboflavin is able to rescue embryogenesis in adult female worms by approximately 50% [39].

If *Wolbachia* are indeed provisioning *B*. *malayi* with metabolites or nutrients, they would require active secretion and transport systems to do so. We determined that the Sec-dependent and Sec-independent systems appear to be constitutively expressed, especially in adult females. We also find genes in the T4SS, responsible for transport across the outer membrane, to be constitutively expressed across all sampled stages with the exception of *wBm0798* in L4. This suggests that the T4SS is not only active in *Wolbachia*, but also important in all stages of development that were included in this study. These results confirm the potential for the filarial dependence on *Wolbachia* products at all stages of the life cycle. Constitutive expression of the heme, phosphate, and lipoprotein ABC transporters is also consistent with the expression of these biosynthetic pathways as phosphate is an essential molecule in nucleotides.

The co-expression network analysis of *Wolbachia* and *B*. *malayi* genes was another approach to define interactions. The use of WGCNA to construct a co-expression network for both *Wolbachia* and *B*. *malayi* genes revealed co-expression of important pathways. A number of resulting modules were significantly correlated with *Wolbachia* density, either positively or negatively. Analysis of GO enrichment of the modules as well as module membership revealed a number of pathways of interest including redox homeostasis and oxidative stress responses as well as the co-expression of DNA repair and replication between the two organisms. Transport mechanisms were also co-expressed, including a nucleoside transporter in *B*. *malayi* co-expressed with *Wolbachia* genes involved in *de novo* purine and pyrimidine biosynthesis. We plan to expand this co-expression network to include additional stages of parasite development, including molting larvae and microfilaria, in order to better represent the dynamics of endosymbiosis over the entire parasitic lifecycle.

In conclusion, our study provides novel insight into the complexity of the interactions between *B*. *malayi* and its endosymbiotic bacteria, *Wolbachia*. We find that it is unlikely that this obligate symbiotic relationship relies on a single process or pathway, but rather on more complex interactions that likely vary over the life cycle of the parasite. This work paves the way for functional validation of the essential role of these associations through the use of RNAi experiments. Elucidation of essential pathways involved in the endosymbiosis between *Wolbachia* and *B*. *malayi* will allow for the identification of novel drug targets.

## Supporting information

S1 Fig MDS of biological replicates. **a**. Clustering of biological replicates using multidimensional scaling analysis of the top 50% most highly expressed *B*. *malayi* genes. **b**. Clustering of biological replicates using multidimensional scaling analysis of the top 50% most highly expressed *Wolbachia* genes. Samples taken during nematode development from L4 to 120 days post infection (dpi), in males (M) and females (F), with a and b denoting separate biological replicates.
(TIF)

S2 Fig qRT-PCR validation. Seven genes (10 pair-wise comparisons) were chosen for confirmation with RT-qPCR. Four of the genes (WSP, Hsp90, life cycle DnaK, and GroEL), with seven pair-wise comparisons, were chosen based on the criteria that they were found to be significantly differentially expressed and had over 50 read counts per stage. Also included are three genes (RibA, HemA, and AfuA) that were found to be constitutively expressed, based on an FDR of 1 in EdgeR. Spearman correlation of 0.987 and a p-value < 2.2e-16.
(TIF)

S1 Table *Brugia* DE genes over female development. Table showing results of pair-wise differential expression analyses between female stages.
(XLSX)

S2 Table *Brugia* DE genes over male development. Table showing results of pair-wise differential expression analyses between female stages.
(XLSX)

S3 Table *Brugia* DE genes between males and females. Table showing results of pair-wise differential expression analyses between male and female stages.
(XLSX)

S4 Table Table of *Wolbachia* DE genes. Table showing results from all pair-wise differential expression analyses for *Wolbachia*.
(XLSX)

S5 Table Module membership assignments from WGCNA. Table showing the module assignments resulting from the hierarchical clustering for *B*. *malayi* and *Wolbachia* genes.
(XLSX)

S6 Table GO term enrichment. Table showing the results of the GO term enrichment analysis for each module, p-value < 0.01.
(XLSX)
