# Supplementary figures and images for: Defining Brugia malayi and Wolbachia symbiosis by stage-specific dual RNA-seq
Source: PLoS Negl Trop Dis. 2017 Mar 30;11(3):e0005357. doi: 10.1371/journal.pntd.0005357 (PMC5373514; doi:10.1371/journal.pntd.0005357)

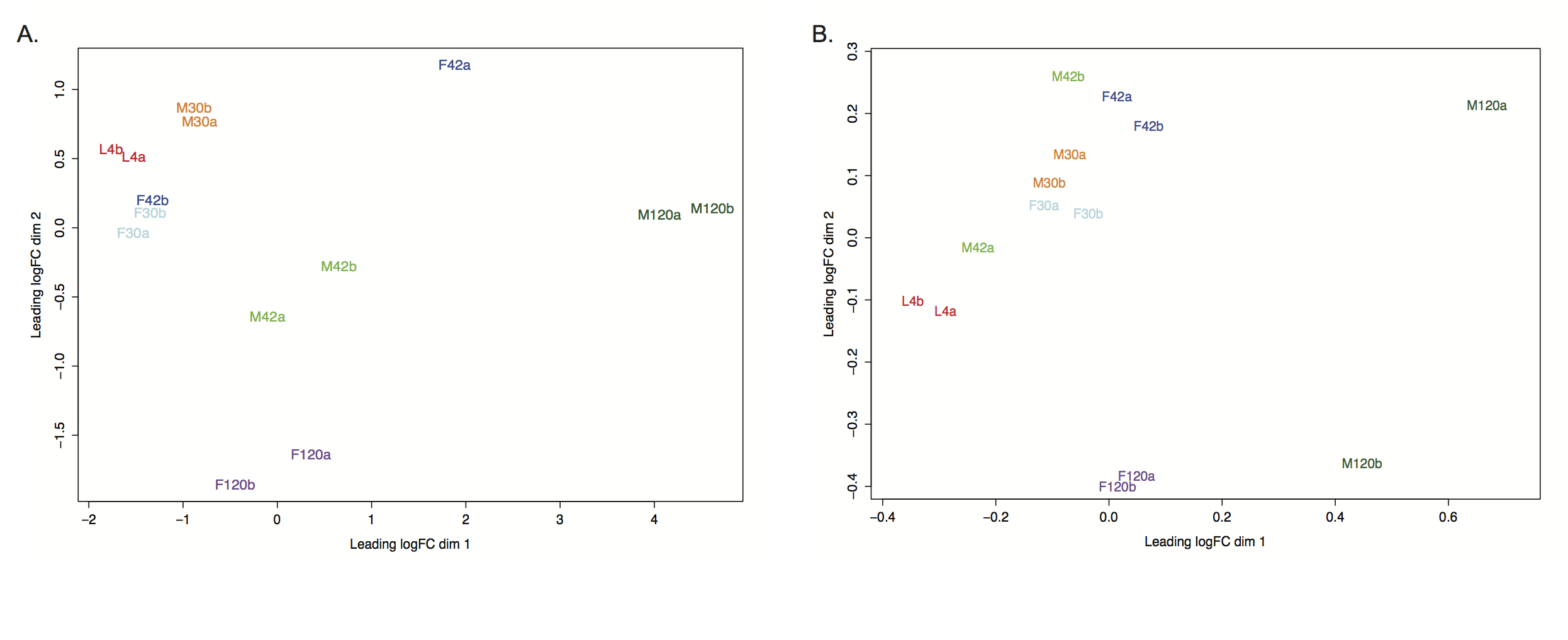

Supplement: S1 Fig — a. Clustering of biological replicates using multidimensional scaling analysis of the top 50% most highly expressed B. malayi genes. b. Clustering of biological replicates using multidimensional scaling analysis of the top 50% most highly expressed Wolbachia genes. Samples taken during nematode development from L4 to 120 days post infection (dpi), in males (M) and females (F), with a and b denoting separate biological replicates. (TIF) [file pntd.0005357.s001.tif]

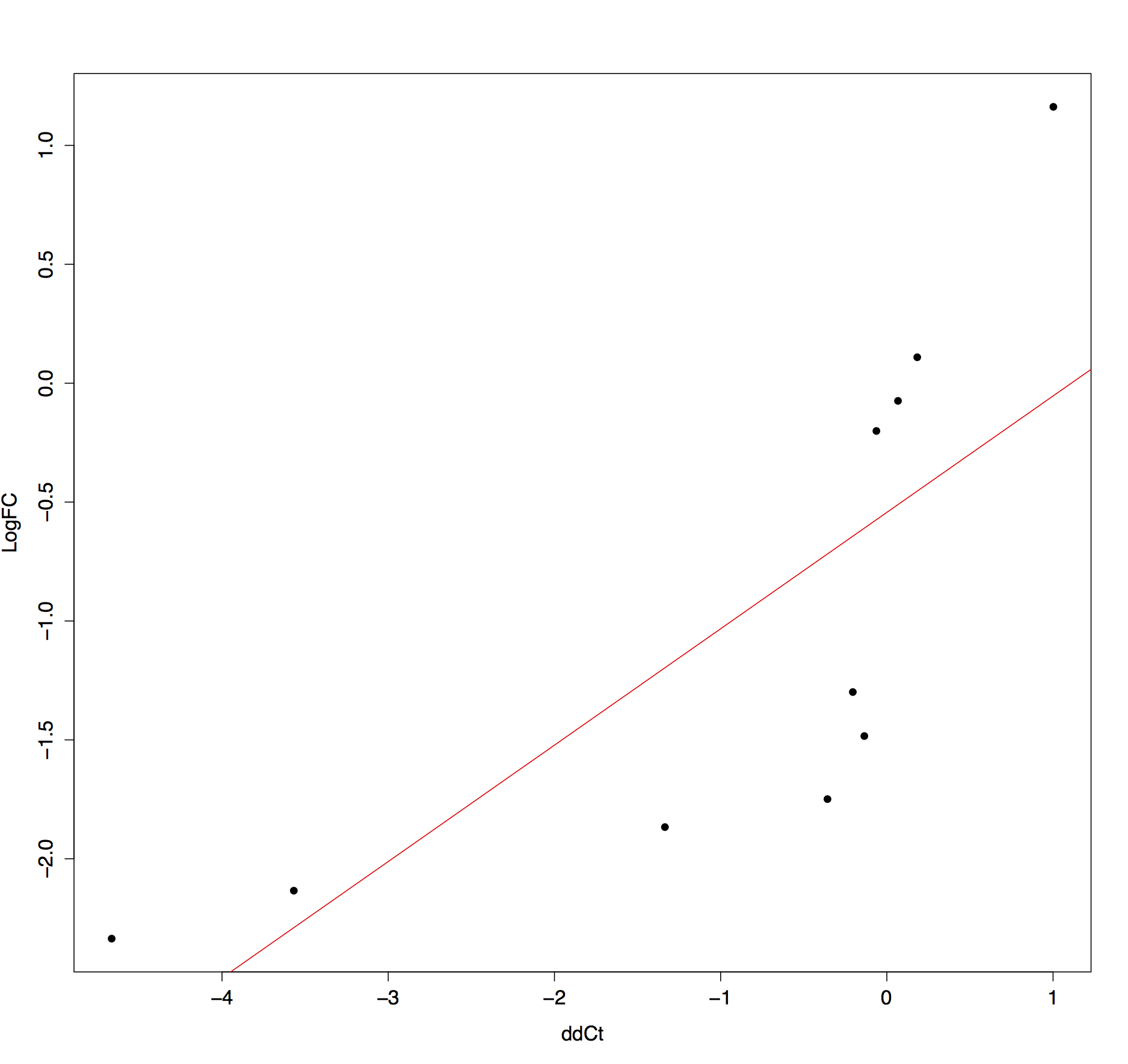

Supplement: S2 Fig — Seven genes (10 pair-wise comparisons) were chosen for confirmation with RT-qPCR. Four of the genes (WSP, Hsp90, life cycle DnaK, and GroEL), with seven pair-wise comparisons, were chosen based on the criteria that they were found to be significantly differentially expressed and had over 50 read counts per stage. Also included are three genes (RibA, HemA, and AfuA) that were found to be constitutively expressed, based on an FDR of 1 in EdgeR. Spearman correlation of 0.987 and a p-value < 2.2e-16. (TIF) [file pntd.0005357.s002.tif]
